# Asymmetry in the Discrimination of Quantity: The Role of Stimulus Generalization

**DOI:** 10.1037/xan0000073

**Published:** 2015-06-29

**Authors:** Richard A. Inman, Robert C. Honey, John M. Pearce

**Affiliations:** 1 School of Psychology, Cardiff University

**Keywords:** discrimination learning, stimulus generalization, quantity discrimination

## Abstract

In order to evaluate 1 account for the asymmetry that has been found with discriminations based on stimulus magnitude, in 5 autoshaping experiments, 2 groups of pigeons received a discrimination between 5 and 20 squares presented on a TV screen. One group received a 20+/5– discrimination, with food signaled by 20 squares but not 5 squares; the other group received the opposite discrimination, 5+/20–. The 20+/5– discrimination was acquired more readily than 5+/20– in Experiments 1, 3a, 3b, and 4. For Experiment 1, the screen was white for the intertrial interval (ITI) and the stimuli were black squares on a white background; for Experiment 3a, the screen was black for the ITI and the stimuli were black squares on a white background; and for Experiments 3b and 4, the screen was white for the ITI and the stimuli were white squares on a black background. In Experiment 2, the stimuli were black squares on a white background, but they were separated by an ITI in which 288 black squares were presented against a white background. The 20+/5– discrimination was now acquired more slowly than the 5+/20– discrimination. The asymmetry in the acquisition of the magnitude discriminations in each experiment is attributed to inhibition being associated with the stimuli present during the ITI. The generalization of this inhibition, along a dimension related to the number of squares on the screen, is then assumed to disrupt the acquisition of 1 discrimination to a greater extent than the other.

A prediction that is common to a number of theories is that a discrimination between two stimuli, in which one signals a significant outcome and the other does not, will be symmetrical. That is, the rate at which the discrimination will be mastered is predicted to be the same, regardless of which stimulus is paired with the outcome (Blough, 1975; Pearce, 1987, 1994; Spence, 1936). In contrast to this prediction, there is a small body of evidence to suggest that discriminations between stimuli of different magnitudes are not symmetrical. These discriminations appear to be acquired more readily when the outcome is paired with the larger rather than the smaller stimulus. The purpose of the present article is to evaluate one explanation for how this asymmetry may be accommodated within current theories of learning.

A recent demonstration of the asymmetry in a magnitude discrimination is provided by Kosaki, Jones, and Pearce (2013) who trained two groups of rats to escape from a square pool of water with four gray walls. Short black panels were attached to one pair of opposing walls, and long black panels were attached to the remaining pair. The long-panel group was required to find a submerged platform beside the long panels, but not the short; while the short-panel group was trained with the platform beside the short, but not the long panels. The discrimination was mastered more successfully by the long-panel than the short-panel group. Indeed, when the lengths of the panels were 50 and 100 cm, then the group required to swim to the short panels showed no evidence of acquiring the discrimination. This asymmetry is not just confined to discriminations based on the geometric properties of objects. A similar effect has been observed when rats receive a discrimination with stimuli of long and short temporal durations in which one, but not the other, signals food. In a number of experiments rats have acquired the discrimination more readily when food was signaled by the long, rather than by the short stimulus (Bouton, & García-Gutiérrez, 2006; Bouton & Hendrix, 2011; Kyd, Pearce, Haselgrove, Amin, & Aggleton, 2008; Todd, Winterbauer & Bouton, 2010). A similar asymmetry has been revealed with discriminations based on the intensity of odors (Pelz, Gerber, & Menzel, 1997) and on the intensity of white noise (Zielinski & Jakubowska, 1977). In these experiments, the discrimination was solved more readily when reward was signaled by the stronger rather than the weaker of the two stimuli.

To explain the asymmetry in discriminations based on the length of objects, Kosaki et al. (2013) developed an account based on the principles of stimulus generalization (see also Mackintosh, 1974, pp. 532–533). Consider the case in which the goal was situated beside the long, but not the short black panels. This arrangement can be expected to result in the long black panels entering into excitatory associations, and the short panels into inhibitory associations (e.g., Spence, 1936). In addition, as a result of their experiences during training, rats are likely to associate regions of the pool, such as the gray walls not covered by the black panels, with the absence of the goal. These background cues, too, may enter into inhibitory associations. If it is accepted that the background cues are more similar to the walls containing the short black panels than those displaying the long black panels, then it follows that the inhibition associated with the background cues will generalize to a greater extent to the walls with short rather than long black panels. Because the short panels also signal the absence of the platform, this generalization is likely to facilitate the acquisition of the discrimination. Now consider the case in which the goal is situated beside the short rather than the long black panels. Generalization of inhibition from the background cues to walls with short black panels will interfere with excitatory conditioning with these panels and disrupt the acquisition of the discrimination. Thus, according to Kosaki et al. (2013), the asymmetry in magnitude discriminations occurs because of inhibition associated with the background cues generalizing to a greater extent to the smaller than the larger of the two discriminative cues.

As they stand, the proposals of Kosaki et al. (2013) should apply to any magnitude discrimination. One purpose of the present article is to test this prediction by examining whether the asymmetry in magnitude discriminations extends to tasks where the variations in the number of objects that are displayed serve as the signals for presence and absence of reward. On the basis of the slender evidence that is available, it appears that discriminations between different quantities or numbers of the same object may well be asymmetrical. In an experiment by Watanabe (1998), pigeons were presented with displays consisting of either two or four red balls with food made available for key pecking in the presence of one of the displays. The two birds who were required to peck for food in the presence of four balls, but not two, each solved the discrimination more readily than the remaining two birds who had to peck in the presence of two balls for food, but not four. For a similar finding from a study using three brown bears see Vonk and Beran (2012). Neither of these experiments was intended as an investigation of whether a discrimination between a large and a small number of the same objects is easier when reward is signaled by the larger rather than smaller number. Moreover, the small sample sizes make it difficult to draw any clear theoretical conclusions from the experiments. Thus the initial purpose of the present experiments was to provide the first direct test of whether an asymmetry exists in discriminations based on different numbers of the same object.

For the first experiment, two groups of pigeons received autoshaping in which the conditioned stimuli (CS) were patterns containing either 5 identical black squares or 20 identical black squares (see Figure 1). The squares were presented against a white background on a TV screen that was illuminated white throughout the intertrial interval (ITI). Food was presented after patterns with 5 squares, CS+, but not after patterns with 20 squares, CS–, for the 5+/20– group. For the 20+/5– group there were 20 squares in CS+ and 5 squares in CS–. According to the proposals of Kosaki et al. (2013), the background cues that are present during the ITI, such as the white TV screen, will enter into inhibitory associations by virtue of being present for prolonged periods in the absence of food. This inhibition will then generalize to the training stimuli, but it is likely that the extent of this generalization will be greater to the pattern containing 5 rather than 20 squares, as the former will contain a larger proportion of background cues than the latter. As a consequence, the generalization of inhibition from background cues is then predicted to facilitate the acquisition of the 20+/5– discrimination, and disrupt the acquisition of the 5+/20– discrimination. The results confirmed this prediction and the purpose of the remaining experiments was, on the one hand, to test further the proposals of Kosaki et al. (2013) and, on the other hand, to gain a preliminary understanding of which aspect of the stimulation present during the ITI was responsible for the asymmetry revealed in Experiment 1.[Fig-anchor fig1]

## Experiment 1

### Method

#### Subjects

The subjects were 16 experimentally naïve adult homing pigeons (*Colomba livia*). They were housed in pairs in a temperature-controlled colony room (approximately 20°C) that was continuously illuminated for 14.5 hours per day, with lights on at 07:00. They had access to water and grit ad libitum but were food deprived and reduced to between 80–85% of their free feeding weights (*M* = 411 g) prior to the start of the experiment. They were maintained at this weight by being fed a restricted diet after each experimental session. They were randomly assigned to the two groups in equal numbers.

#### Apparatus

Eight operant chambers (30.0 × 33.0 × 35.0 cm, L × W × H) were used. Each chamber was constructed from three aluminum walls, an aluminum ceiling, and a clear acrylic door serving as the fourth wall. A wire mesh served as the floor for these chambers. A tray lined with absorbent, odor—removing paper served to collect waste below the mesh. The left-hand wall looking into the chambers contained a clear acrylic response key (8.3 cm × 6.3 cm), which was hinged at the top. The midpoint of this key was 24 cm from the chamber floor and situated halfway between the two side walls. Pecks on this panel were detected by a reed relay, which was operated whenever a magnet on the bottom of the key was displaced by a distance greater than 1 mm. A color, thin-film transistor TV (Saka; 15.5 × 8.7 cm) was used to present the stimuli. Food was delivered into a food well (4.6 × 5.4 cm) which was located in the same wall. The midpoint of the entrance to this food well was 9.0 cm from the chamber floor and 7.0 cm to the left of the midline of the wall. Conditioning seed (Bucktons®) was made available inside the food well via a grain feeder (Colbourn Instruments, Lehigh Valley, PA). A PC with Whisker software, and programmed in Visual Basic 6.0, controlled the experimental events and recorded the number of pecks made on the key. Each chamber was contained in an individual sound-attenuating chamber which was shut during the experimental session. Throughout each experimental session test chambers were illuminated by a single 2.8-W bulb, operated at 24 V and located in the chamber ceiling.

#### Procedure

The subjects first received six sessions of pretraining, each lasting 60 min, in which they were trained to retrieve food from the food well whenever the grain feeder was operated. The televisions behind the response keys were turned off for this stage of the experiment. In order to encourage them to attend to the TV screen, the birds then received 13 sessions of autoshaping in which a colored cross was presented on the screen against a background of a different color. For half the birds in each group the cross was red and the background was green, whereas for the remainder the cross was green and the background was red. The cross was 40 mm wide and 40 mm high with lines that were 7 mm thick. The cross was presented for 10 s and food was presented for 4 s as soon as the cross was removed from the screen. Throughout the ITI, the entire TV screen was the same color as the background for the conditioning trials. The duration of the ITI was increased gradually from 40 to 60 s during this stage.

Throughout the following eight sessions, the TV screen was entirely white during every ITI. For each conditioning trial the screen was again white but also displayed either 5 or 20 black squares (3 mm × 3 mm). The squares were arranged randomly within a notional circle of diameter 4.1 cm, the center of which was coincident with the center of the TV screen. There were 10 different variants of the 5-square and the 20-square stimuli. The stimuli were presented in a random sequence with the constraint that no more than two trials with the same numbers of squares could occur in succession. The duration of each stimulus was 10 s, and the ITI was 40, 60, or 80 s (*M* = 60 s) determined randomly for each trial. There were 20 trials with each of the two stimuli within each session. Food was not presented after any stimulus during the first two sessions of this stage. The purpose of these extinction sessions was to reduce responding during the experimental stimuli to a low rate, and thereby make it possible to observe differences in the acquisition of the discrimination when one of the stimuli, but not the other signaled food during the remaining six sessions of the experiment. During the final six sessions, every presentation of a 5-square stimulus, but not a 20-square stimulus, was followed by the delivery of food for 4 s for the 5+/20– group, whereas food was presented after the 20-square but not the 5-square stimulus for the 20+/5– group.

#### Data analysis

Individual mean rates of responding, in responses per min, were recorded for all trials in the final extinction session, and for each of the six sessions of discrimination training. The rates of responding during an interval of 10-s before every trial were also recorded. The analysis of these results was conducted with analyses of variance (ANOVAs) using a rejection criterion of *p* < .05. The reported effect size for ANOVAs with more than one factor is partial eta-squared (η_p_^2^), while for comparisons between two means it is eta squared (η^2^). For both measures of effect size, 95% confidence intervals (CI) were computed using the method reported by Steiger (2004). When the majority of response rates were at zero, or close to zero, which was the case for the data analyzed in the final extinction session, and those recorded during the pre-CS intervals, then nonparametric statistical tests were used.

### Results

The mean rates of responding to the 5-square and 20-square stimuli during the second session of extinction training were very low. For Group 5+/20– the mean rate of responding to the 5-square stimuli was 0.9 responses per min and to the 20-square stimuli it was 1.5 responses per min. The equivalent results for the 20+/5– group were, respectively, 1.1 and 1.0 responses per min. The difference between the rates of responding to the 5- and 20-square stimuli was not significant in either group, Wilcoxon’s *z*s(6) < 1.16, *p*s > .10. A between-groups comparison of the mean rates of responding to both stimuli combined also revealed a nonsignificant difference, *U*(8, 8) = 23, *p* > .10.

Figure 2 shows the group mean rates of responding by the two groups during the reinforced, CS+, and nonreinforced, CS–, stimuli, and during the pre-CS intervals, for the six sessions of discrimination training. The discrimination was acquired more readily by the 20+/5– than the 5+/20– group. These observations were supported by a three-way ANOVA of individual mean rates of responding during CS + and CS– for each of the six sessions, which revealed a significant, Stimulus × Group interaction, *F*(1, 14) = 6.07, *p* = .027, η_p_^2^ = .30, 95% CI [.00, .57]. The remaining findings from the ANOVA were a significant effect of stimulus, *F*(1, 14) = 29.38, *p* < .001, η_p_^2^ = .68, 95% CI [.29, .81], and session, *F*(5, 70) = 6.19, *p* < .001, η_p_^2^ = .31, 95% CI [.10, .42], but not group, *F* < 1. The interactions of Session × Group, *F*(5, 70) = 4.0, *p* = .003, η_p_^2^ = .22, 95% CI [.03, .33], and Stimulus × Session, *F*(5, 70) = 21.90, *p* < .001, η_p_^2^ = .61, 95% CI [.43, .69], were significant, but the three-way interaction, *F*(5, 70) = 2.20, *p* = .064, was not significant. Tests of simple main effects based on the Stimulus × Group interaction revealed a significant difference in the rates of responding to CS+ and CS– for the 20+/5– group, *F*(1, 14) = 31.08, *p* < .001, η_p_^2^ = .69, 95% CI [.31, .81], but not the 5+/20– group, *F*(1, 14) = 4.37, *p* = .055.[Fig-anchor fig2]

Figure 2 also shows that pigeons made few responses on the illuminated key during the 10-s pre-CS period. A between-groups comparison of individual mean rates of responding for the six sessions combined revealed that they were not significantly different, *U*(8, 8) = 26, *p* > .10.

### Discussion

The more rapid acquisition of the discrimination by the 20+/5– group than the 5+/20– group confirms that discriminations in which reward and nonreward are signaled by different numbers of identical objects are asymmetrical. Moreover, in keeping with discriminations involving stimuli that differ either in physical length, or temporal duration, the asymmetry favored the discrimination in which reward was signaled by the stimulus that is of larger rather than smaller magnitude. We noted in the Introduction that the asymmetry may be a consequence of the generalization of inhibition from cues present during the ITI to the cues used to signal the delivery and absence of food. If it is accepted that the extent of this generalization is greater to the smaller than the larger of the two cues, then it would follow that the 20+/5– discrimination will benefit the generalization of inhibition from the ITI cues, whereas the 5+/20–discrimination will be disrupted by this generalization. Experiment 2 was conducted in order to evaluate this explanation for the results from Experiment 1.

## Experiment 2

The experiment contained two groups who received the same discrimination as their namesakes in Experiment 1. In contrast to the first experiment, the screen during the ITI was not uniformly white but, for both groups, it consisted of a white background with 288 black squares randomly distributed over the entire screen (see Figure 3). These squares were identical to those used to create the experimental stimuli. In terms of the proposals put forward by Kosaki et al. (2013), the screen displaying a large number of squares during the ITI will enter into an inhibitory association, the effects of which will generalize to the experimental stimuli. The extent of this generalization is likely to be greater to the stimulus comprising 20 squares than the one comprising 5 squares. As a consequence, the influence of the inhibition associated with the cues present during the ITI will be to promote the acquisition of the 5+/20– discrimination, by augmenting the effects of the nonreinforced trials with the 20-square stimulus, and disrupt the acquisition of the 20+/5– discrimination, by counteracting the effects of excitatory conditioning with the 20-square stimulus. Thus the proposals of Kosaki et al. (2013) again predict there will be an asymmetry in the acquisition of the discriminations by the two groups but, on this occasion, performance will be superior by the group for which food is signaled by the 5-square rather than the 20-square stimulus.[Fig-anchor fig3]

### Method

#### Subjects, apparatus and procedure

The subjects were 16 experimentally naïve homing pigeons with a mean free-feeding weight of 471 g. Their housing, and the method of food deprivation was the same as for Experiment 1. The birds were assigned at random to the two groups at the start of the experiment. The apparatus was the same as for Experiment 1.

Pretraining was the same as for Experiment 1, except that there were four sessions of magazine training and five sessions of autoshaping. For each of the final nine sessions, the TV screen during every ITI was a white background covered with 288 squares. The squares were identical to those used to create the test patterns and were spread with approximately equal spacing but randomly distributed across the entire screen. The same pattern of 288 squares was used for every ITI. The experimental stimuli presented after each ITI consisted of patterns comprising either 5 or 20 black squares against a white background. These patterns were not followed by food for three extinction sessions. For the remaining six sessions, patterns with 5 squares were followed by food, and patterns with 20 squares were not followed by food for the 5+/20– group, whereas for the 20+/5– group food was presented after patterns with 20 but not 5 squares. Procedural details that have been omitted were the same as for Experiment 1.

### Results

One pigeon from the 20+/5– group failed to respond during any session of discrimination training and was therefore excluded from the experiment. The average rates of responding per min to stimuli during the final session of extinction was low for all groups. For the 20+/5– group the mean rate of responding to the 20-square stimuli was 3.0 responses per min and 1.9 responses per min to the 5-square stimuli. This difference was not significant, Wilcoxon’s *z*(6) = .11, *p* > .10. For the 5+/20– group the rates of responding were 2.0 and 2.3 responses per minute for the 20- and 5-square stimuli respectively. Again, this difference was not significant, Wilcoxon’s *z*(7) = 1.69, *p* > .10. Between group comparisons of the mean rates of responding to both stimuli also revealed a nonsignificant difference, *U*(7, 8) = 26.5, *p* > .10.

The results from the six sessions of discrimination training can be seen in Figure 4, which shows that the 5+/20– group acquired its discrimination more readily than the 20+/5– group. Indeed, the latter group showed no sign of mastering the discrimination, even after six sessions of training. A three-way ANOVA with the factors of group, session and stimulus (CS + vs. CS–) confirmed these observations revealing a significant three-way interaction, *F*(5, 65) = 7.92, *p* < .001, η_p_^2^ = .38, 95% CI [.15, .49], and significant Group × Stimulus interaction, *F*(1, 13) = 10.32, *p* = .007, η_p_^2^ = .44, 95% CI [.05, .67]. The remaining findings were a significant effect of session, *F*(5, 65) = 26.70, *p* < .001, η_p_^2^ = .67, 95% CI [.51, .74], and Stimulus × Session interaction, *F*(5, 65) = 8.25, *p* < .001, η_p_^2^ = .39, 95% CI [.16, .50], but no significant effect of group, *F*(1, 13) = 1.82, *p* < .10, and no Group × Session interaction, *F* < 1. Tests of simple main effects based on the significant three-way interaction revealed a significant Group × Stimulus interaction from session three onward, *F*s(1, 78) > 4.26, *p* < .043, η_p_^2^ = .05, 95% CI [.00, .17]. In addition, this analysis revealed a significant effect of stimulus from session three onward for the 5+/20– group, *Fs*(1, 78) > 6.36, *p*s < .014, η_p_^2^ = .08, 95% CI [.00, .20], but no effect of stimulus at any session for the 20+/5– group, *F*s < 1.[Fig-anchor fig4]

Figure 4 also shows that throughout the final six sessions responding during the pre-CS periods was at a very low rate for both groups. A comparison of individual mean rates of responding for the six sessions combined revealed that they were not significantly different, *U*(7, 8) = 14, *p* > .10.

### Discussion

The results from the two groups were as anticipated. Thus the group for whom 5 squares signaled food, and 20 squares signaled the absence of food, readily acquired the discrimination whereas this was not the case for the group for whom 20 squares signaled food, and 5 squares signaled the absence of food. This pattern of results strongly suggests that one cause of the asymmetry in magnitude discriminations is the nature of the stimulation present during the ITI. If this stimulation is more similar to the small than the large training stimulus then the discrimination with the small stimulus as a signal for reward will be acquired with more difficulty than when the large stimulus signals reward. On the other hand, when the stimulation during the ITI is more similar to the large than the small stimulus, then the discrimination with food signaled by the small rather than the large stimulus will be acquired more readily. It is worth remarking that the present results are the first occasion on which the asymmetry in a magnitude discrimination has favored training in which reward is signaled by the smaller, rather than the larger of the two stimuli.

An unexpected finding from the experiment was the failure of the 20+/5– group to show any indication of solving its discrimination. One might argue that this failure occurred because the group was unable to tell the difference between the training stimulus with 20 squares that signaled food, and the pattern of stimulation that was present during the ITI. Such an explanation, however, is challenged by the finding that throughout the experiment responding during the ITI was extremely slow, and consistently slower than during either of the training stimuli. Some other explanation is thus needed in order to explain the failure of the 20+/5– group to solve the discrimination. Apart from suggesting that insufficient sessions were administered, we are at a loss to suggest what this explanation might be.

## Experiment 3a

**Figure 1 fig1:**
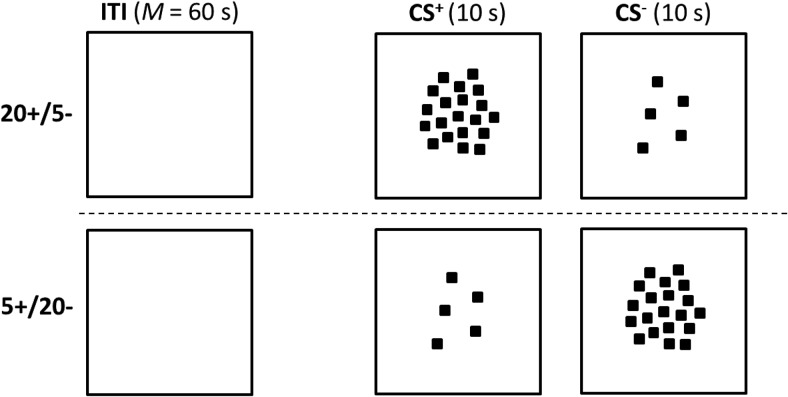
The stimuli for Experiment 1 (the figure is for illustrative purposes and does not depict accurately the images displayed to the pigeons).

**Figure 2 fig2:**
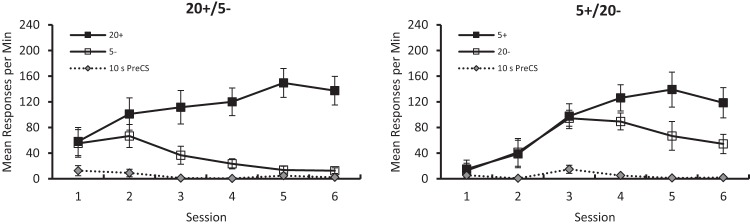
The mean rates of responding to conditioned stimuli (CS) CS+ and CS– for the six sessions of training for the 20+/5– (left-hand panel), and 5+/20– (right-hand panel) groups of Experiment 1. Error bars represent ± *SEM.*

**Figure 3 fig3:**
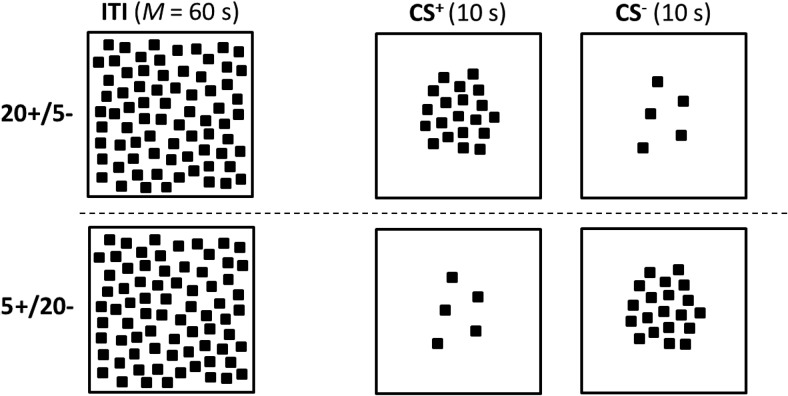
The stimuli for Experiment 2 (the figure is for illustrative purposes and does not depict accurately the images displayed to the pigeons).

**Figure 4 fig4:**
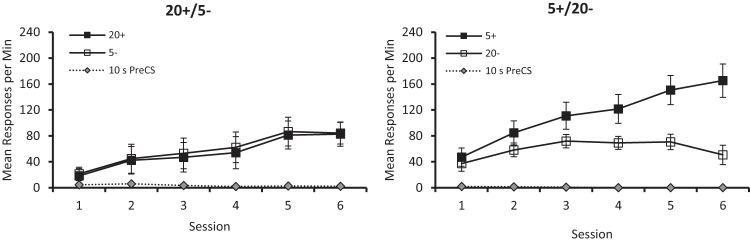
The mean rates of responding to conditioned stimuli (CS) CS+ and CS– across the six sessions of training for the 20+/5– (left-hand panel) and 5+/20– (right-hand panel) groups of Experiment 2. Error bars represent ± *SEM*.

The results from Experiment 2 point to the importance of the stimuli present during the ITI for the asymmetry between the acquisition of a 5+/20– and a 20+/5– discrimination. We have argued that this asymmetry stems from generalization between the cues present during the ITI and those present during trials with CS+ and CS–. The purpose of the present experiment was to identify the dimension along which this generalization takes place. Inspection of Figures 1 and 3 reveals that generalization could be based on at least two possible dimensions. One dimension will be referred to as brightness. In Experiment 1 the overall brightness of the screen on which the stimuli were displayed was maximal during the ITI, not quite so bright for the trials with five squares, and least bright during trials with 20 squares. On this basis there was more scope for generalization of inhibition from the ITI to the patterns with 5 rather than 20 squares, which would then account for the asymmetry that was observed. In Experiment 2, the numerous squares present during the ITI would mean that the screen was at its darkest during these intervals, and any inhibition associated with it would generalize to a greater extent to patterns with 20 rather than 5 squares, and result in the opposite asymmetry to that seen in Experiment 1.

The second possible dimension will be referred to as number. In Experiment 1, there were no squares present during the ITI, and either 5 or 20 squares present during the conditioning trials. If it is accepted that the absence of squares serves as an anchor representing zero on the dimension of number, then it follows there will be more generalization of inhibition from the ITI to the 5-square than the 20-square patterns in Experiment 1. These differences in generalization of inhibition will then disrupt the 5+/20– discrimination and facilitate the 20+/5– discrimination. Conversely, there will be more scope for the generalization of inhibition from the ITI patterns composed of 288 squares to the 20-square than 5-square patterns and thus the 20+/5– discrimination will be disrupted to a greater extent than the 5+/20– discrimination. By appealing to either the dimension of brightness or number, therefore, it is possible to explain the results thus far. The purpose of the present experiment was to identify which of these dimensions was used to solve the discriminations.

**Figure 5 fig5:**
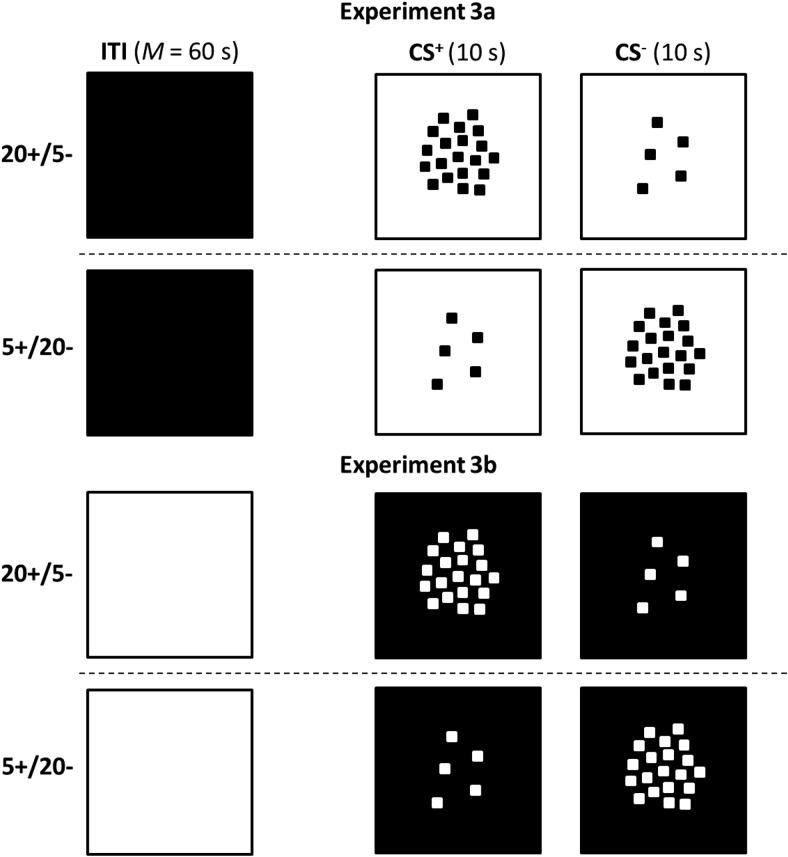
The stimuli for Experiments 3a and 3b (the figure is for illustrative purposes and does not depict accurately the images displayed to the pigeons).

The two groups of Experiment 3 received the same discrimination training as for the previous experiments, except that the TV screen was entirely black during the ITI (see Figure 5). If generalization between the experimental stimuli and the cues present during the ITI is based on the dimension of brightness, then there will be more scope for generalization between the black TV screen of the ITI and the patterns containing 20 black squares, than the patterns containing 5 black squares. On this basis, therefore, the proposals of Kosaki et al. (2013) predict that a group receiving a 5+/20– discrimination will acquire it more readily than one receiving a 20+/5– discrimination. In other words, using a black screen during the ITI should reverse the asymmetry that was seen in Experiment 1, in much the same manner as the 288 black squares that were presented on the white screen during the ITI in Experiment 2. A different outcome is predicted if generalization between the stimuli used in the experiment is based on number. The absence of any small squares on the black screen during the ITI might result in it being treated as zero on the dimension of number and result in more generalization of inhibition from this cue to the patterns displaying 5 rather than 20 squares. As a consequence, despite the very different stimulation provided by the TV screen during the ITI in the present experiment, and Experiment 1, the outcome of both experiments is predicted to be the same. The 20+/5– group should acquire its discrimination more readily than the 5+/20– group.[Fig-anchor fig5]

### Method

#### Subjects, apparatus and procedure

Sixteen experimentally naïve pigeons with a mean free-feeding weight of 485 g were used. They were from the same stock and housed in the same manner as for Experiment 2. The method of food deprivation, the apparatus, and the procedural details concerning pretraining were the same as for Experiment 2.

The pretraining was followed by three sessions of extinction and six sessions of discrimination training in which the pigeons were presented with the same 20- and 5-square stimuli as in Experiment 2. For the duration of each ITI the screen was entirely black. In all other aspects the procedural details were identical to Experiment 2.

### Results

The average rate of responding per minute to stimuli during the final session of extinction was low for all groups. For Group 20+/5– the mean rate of responding to the 20-square stimuli was 1.3 responses per min and 2.1 responses per min to the 5-square stimuli. This difference was not significant, Wilcoxon’s *z*(4) = 1.84, *p* > .05. For Group 5+/20– the equivalent rates of responding were 1.0 and .5 responses per minute, respectively. Again this difference was not significant, Wilcoxon’s *z*(7) = 1.27, *p* > .10. Between group comparisons of the mean rates of responding to both stimuli also revealed a nonsignificant difference, *U*(8, 8) = 17.5, *p* > .10.

**Figure 6 fig6:**
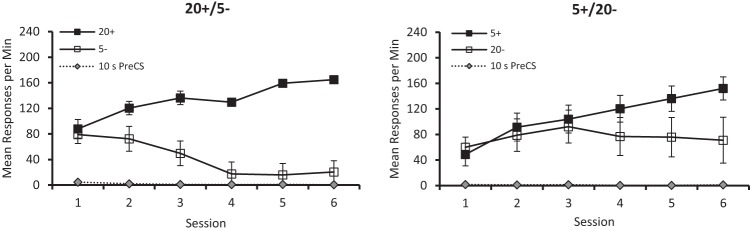
The mean rates of responding to conditioned stimuli (CS) CS+ and CS– for the six sessions of training for the 20+/5– (left-hand panel) and 5+/20– (right-hand panel) groups of Experiment 3a. Error bars represent ± *SEM*.

The results from the six sessions of discrimination training can be seen in Figure 6, which shows that the 20+/5– group acquired its discrimination more readily than the 5+/20– discrimination. In support of this observation, a three-way ANOVA with the factors of group, session and stimulus (CS + vs. CS–) revealed a Group × Stimulus interaction, *F*(1, 13) = 10.29, *p* = .006, η_p_^2^ = .44, 95% CI [.05, .67]. The three-way interaction fell short of the accepted level of significance, *F*(5, 70) = 2.21, *p* = .063, η_p_^2^ = .14, 95% CI [.00, .24]. The remaining findings were a significant effect of session, *F*(5, 70) = 6.95, *p* < .001, η_p_^2^ = .33, 95% CI [.12, .44], a significant Stimulus × Session interaction, *F*(5, 70) = 28.46, *p* < .001, η_p_^2^ = .67, 95% CI [.51, .74] and a significant Group × Session interaction, *F*(5, 70) = 5.56, *p* < .001, η_p_^2^ = .28, 95% CI [.08, .40] but no significant effect of group, *F* < 1. Tests of simple effects on the significant Group × Stimulus interaction revealed a significant effect of stimulus for the 20+/5– group, *F*(1, 14) = 50.84, *p* < .001, η_p_^2^ = .78, 95% CI [.46, .87] and the 5+/20– group, *F*(1, 14) = 6.73, *p* = .021, η_p_^2^ = .32, 95% CI [.00, .58]. There was no significant effect of group for either the CS + or CS–, *F*s(1, 28) < 1.66, *ps* > .10.[Fig-anchor fig6]

Figure 6 also shows that responding during the pre-CS periods was at a very low rate for both groups throughout the final six sessions. A comparison of individual mean rates of responding for the six sessions combined revealed that they were not significantly different for the two groups, *U*(8, 8) = 30, *p* > .10.

### Discussion

Despite the TV screen being black during the ITI of the present experiment, as compared with white for Experiment 1, the results from both studies were remarkably similar. The 20+/5– discrimination was acquired more readily than the 5+/20– discrimination. If this asymmetry in the acquisition of the two discriminations is a consequence of generalization of inhibition from the ITI being greater to one pattern than the other, then the present results indicate that the dimension along which generalization takes place is unlikely to be brightness. For reasons noted in the introduction to the experiment, generalization of inhibition along this dimension would result in the opposite pattern of results to that obtained. In contrast, provided it is accepted that a blank screen that is entirely black represents zero small squares, then the above results can be explained by assuming that generalization took place along the dimension of number. A greater amount of inhibition that would then generalize from the ITI to patterns with 5, rather than 20 squares, and thus favor the 20+/5– over the 5+/20– discrimination.

## Experiment 3b

The purpose of Experiment 3b was to test the theoretical conclusions that were drawn from the previous experiment. Training was similar to that for Experiment 3a, but the screen was entirely white during the ITI, and the patterns consisted of white squares on a black background (see Figure 5). If birds rely on the dimension of number to solve the discrimination then a similar result to that observed in Experiments 1 and 3a will be found. The absence of squares during the ITI will ensure that the value of no squares will enter into an inhibitory association, which will generalize more strongly to the pattern displaying 5 rather than 20 squares and thereby help the 20+/5– discrimination and hinder the 5+/20– discrimination. On the other hand, if birds rely on the dimension of brightness to solve the discrimination, the high level of brightness of the screen during the ITI will enter into an inhibitory association. The effects of this association will then generalize more strongly to patterns with 20 squares than with 5 squares, because of the greater brightness of the former than the latter, and result in 20+/5– discrimination being harder to acquire than the 5+/20– discrimination.

### Method

#### Subjects, apparatus and procedure

The subjects were 16 experimentally naïve, adult homing pigeons with a mean free-feeding weight of 422 g. They were food deprived and housed in the same manner as for Experiment 1. At the start of the experiment they were randomly assigned to two groups. The apparatus was the same as for Experiment 1.

The pretraining was the same as for Experiment 1, except that there were seven sessions of magazine training and six sessions of autoshaping. Both groups then received two sessions of extinction training and then six sessions of discrimination training. For these final eight sessions the screen was white during the ITI and black with either 5 or 20 white squares for the training trials. Procedural details that have been omitted were the same as for Experiment 1.

### Results

The average rate of responding per minute to both types of stimuli on the final day of extinction was low for all groups. For the 20+/5– group the mean rate of responding to the 20-square stimuli was .5 responses per min and .3 responses per min to the 5-square stimuli. This difference was not significant, Wilcoxon’s *z*(4) = 1.13, *p* > .10. For the 5+/ 20– group the equivalent values were .3 and .4 responses per min respectively. Again, this difference was not significant, Wilcoxon’s *z*(4) = .68, *p* > .10. Between group comparisons of the mean rates of responding to both stimuli also revealed a nonsignificant difference, *U*(8, 8) = 24.5, *p* > .10.

**Figure 7 fig7:**
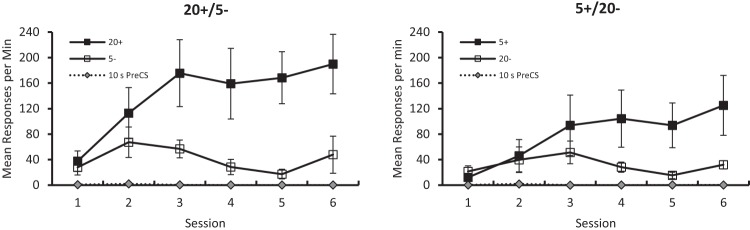
The mean rates of responding to conditioned stimuli (CS) CS+ and CS– across the six sessions of training for the 20+/5– (left-hand panel) and 5+/20– (right-hand panel) groups of Experiment 3b. Error bars represent ± *SEM.*

The results from discrimination stage can be seen in Figure 7, which shows both groups were able to discriminate successfully between visual arrays consisting of 5 and 20 white squares on a black background. It is also evident from the figure that the 20+/5– discrimination was acquired more readily than the 5+/20– discrimination.[Fig-anchor fig7]

A three-way ANOVA with factors of group, stimulus and session confirmed that overall responding to the reinforced stimuli was significantly faster than to the nonreinforced stimuli, *F*(1, 14) = 13.72, *p* = .002, η_p_^2^ = .49, 95% CI [.09, .70]. However, the critical Stimulus × Group, *F*(1, 14) = 1.69, *p* = .21, and Stimulus × Group × Session *F* < 1, interactions were not significant. The analysis also revealed a significant effect of session, *F*(5, 70) = 11.31, *p* < .001, η_p_^2^ = .45, 95% CI [.24, .55], and a significant Stimulus × Session interaction, *F*(5, 70) = 9.71, *p* < .001, η_p_^2^ = .41, 95% CI [.20, .51]. The effect of group, *F*(1, 14) = 1.17, *p* = .30, and the Group × Session, *F* < 1, interaction were not significant. A separate analysis conducted on individual mean rates of responding during the 10-s pre-CS periods for the six sessions combined. The analysis revealed that the slow rates of responding during these periods were not significantly different between groups, *U*(8, 8) = 24.5, *p* > .10.

**Figure 8 fig8:**
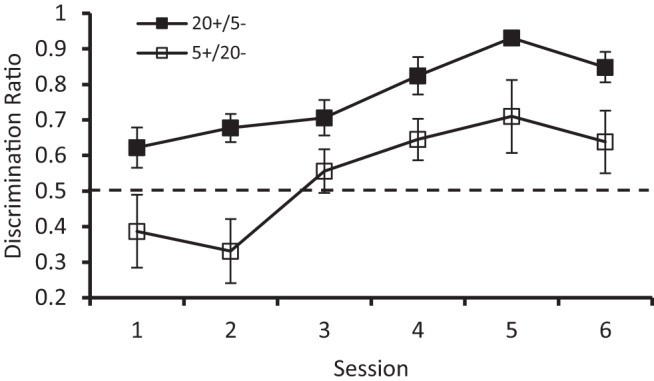
Discrimination ratios for the 20+/5– and 5+/20– groups of Experiment 3b. Error bars represent ± *SEM.*

Inspection of Figure 7 reveals that the error bars for the reinforced trials in both groups were unusually large, when compared with those from the previous experiments. To take account of this variability in individual rates of responding, the raw data were transformed into discrimination ratios calculated in the following way: the mean rates of responding on reinforced trials (CS+), *A,* and nonreinforced trials (CS–), *B*, was calculated for every session for every bird. A ratio of the form *A*/(*A + B*) was then calculated. A ratio greater than .50 indicates that a pigeon was pecking more rapidly to CS+ than CS–. Figure 8 presents the group mean ratios for each of the six sessions of discrimination training and shows that the discrimination was mastered more successfully by the 20+/5– group than the 5+/20– group. In support of this observation, analysis of the discrimination ratios with a two-way ANOVA revealed significant effects of group, *F*(1, 14) = 10.60, *p* = .006, η_p_^2^ = .43, 95% CI [.05, .65]. There was also a significant effect of session, *F*(5, 70) = 12.47, *p* < .001, η_p_^2^ = .47, 95% CI [.26, .57] but the interaction was not significant, *F* < 1.[Fig-anchor fig8]

### Discussion

The results from the experiment are entirely consistent with the claim that pigeons relied on information about the number of squares on the screen in order to solve the discriminations. The asymmetry that was observed across discrimination training can be understood if generalization of inhibition was based on information about the number of squares on the screen: zero, five, or 20. At the same time, the pattern of results is opposite to that predicted if stimulus generalization was based on the overall illumination of the screen

In contrast to the previous experiments, the analysis of the rates of responding during the reinforced and nonreinforced stimuli of the training stage failed to reveal a significant interaction with the effect of group. From Figure 7 it is evident that numerically 20+/5– discrimination was acquired more readily than the 5+/20– discrimination. It is also evident from the error bars in the figure that the within-group variation of response rates was considerable, which might explain the failure on this occasion to obtain a significant interaction involving the factors of group and stimulus. In support of this argument we can note that when the results were analyzed in terms of discrimination ratios, in order to reduce the within-group variation in the data, then a significant asymmetry in the acquisition of the discriminations by the two groups was observed. In view of this outcome analyses of the relevant discrimination ratios in Experiments 1, 2, and 3 were conducted, and each of them revealed a significant asymmetry in the acquisition of the different discriminations. A surprising aspect of the discrimination ratios plotted in Figure 8 is that for the first two sessions, the mean ratios were less than .50 for the 5+/20– group. This effect is a consequence of several birds in this group failing to respond on any conditioning trial and thus resulting in them being assigned a score of 0.

## Experiment 4

The asymmetry revealed in each of the previous experiments has been explained by assuming there was generalization of inhibition from the ITI to the experimental stimuli along the dimension of number. This conclusion then raises the question of how the dimension of number should be conceptualized. The dimension could be concrete and thus tied closely to the physical properties of objects that differ in number. Such a conceptualization would result in generalization from one quantity to another, but only when the objects in the two quantities are identical. Alternatively, the dimension could be more abstract and represent the number of squares, without regard to their physical properties (e.g., Dumont, Jones, Pearce, & Kosaki, 2015). According to this proposal, generalization based on number might take place even when there is a change in a feature of the objects, such as their color (black or white). In order to choose between these possibilities, the pigeons from Experiment 1 were used for one further experiment. The experiment commenced with a period in which the two groups of that experiment continued with their original training: 5+/20– and 20+/5–. The TV screen was therefore white for the ITI, and white with black squares for the experimental stimuli. After this training the birds received a new discrimination for which the screen was again white for the ITI, but it was now black with either five or 20 white squares for the experimental stimuli. Thus the patterns were the negatives of those used for the initial training and were similar to those shown in the lower half of Figure 5. If the original discrimination was based on the concrete properties of the black squares, then the effects of the training with the original stimuli will not transfer to the white squares of the new discrimination. On the other hand, if the representation of the number of squares on the screen is more abstract, then transfer from the old to the new discrimination may well take place.

In order to evaluate these predictions, the two original groups were divided into four groups. The 5+/20–/ Same group was composed of four birds from the original 5+/20– group and received a 5+/20– discrimination with the new stimuli. Likewise, the 20+/5–/Same group was composed of four birds from the original 20+/5– group and received a 20+/5– discrimination with the new stimuli. The remaining two groups received the opposite training in the final stage to that administered originally. The 20+/5–/Diff group initially received a 5+/20– discrimination but was then given a 20+/5– discrimination, while the 5+/20–/Diff group received a 20+/5– discrimination followed by 5+/20–. If the original discrimination was solved by learning about the significance of different numbers of black squares, then performance on the new discrimination should not be influenced by the effects of the original training. On this basis, the 20+/5–/Same and the 20+/5–/Diff groups should perform similarly on the new discrimination, and so too should the 5+/20–/Same and the 5+/20–/Diff groups. If, however, the original training resulted in the number of squares being represented in a more abstract fashion, then the acquisition of the new discrimination should be affected by the original training. That is, the two groups receiving the same discrimination in both stages would be expected to acquire the new discrimination more rapidly than the groups receiving different discriminations.

Whatever the fate of the foregoing predictions, a further prediction concerning the experiment is that the performance of the groups receiving the 20+/5– discriminations in the final stage will be superior to groups receiving the 5+/20– discriminations. Given that the screen was entirely white during the ITI, the absence of any small squares would be expected to enter into an inhibitory association. Generalization of this inhibition would then disrupt the acquisition of the 5+/20– discrimination to a greater extent than the 20+/5– discrimination, and result in the former being acquired more slowly than the latter.

### Method

#### Subjects, apparatus and procedure

The subjects were the same 16 pigeons used in Experiment 1. The apparatus was the same as for Experiment 1.

For Stage I, the birds received six sessions of discrimination training, the details of which were the same as for Experiment 1. At the outset of this training, each of the two groups from Experiment 1was divided at random into two groups with four birds in each group. On the day following the completion of Stage I, the four groups received six sessions of training in Stage II. The ITI was again white but, during the trials, white squares were presented against a black background. The 20+/5–/Same group and the 5+/20–/Same group received the same discrimination in Stage II that they were given in Stage I, whereas the 20+/5–/Diff group and 5+/20–/Diff groups received, respectively, a 20+/5– and a 5+/20– discrimination in Stage II and the opposite discrimination in Stage I. Procedural details that have been omitted were the same as for Experiment 1

### Results

All four groups showed consistently faster responding to CS + than CS– throughout the six sessions of training in Stage I. The mean rates of responding to CS + across the six sessions were 129.4 (*SE* = 38.5), 170.2 (*SE* = 31.5), 142.0 (*SE* = 42.0) and 159.3 (*SE* = 28.3) responses per minute for the 20+/5–/Same, 5+/20–/Same, 20+/5–/Diff and 5+/20–/Diff groups respectively. The mean rates of responding to CS– for the same groups were 11.8 (*SE* = 1.0), 30.1 (*SE* = 10.7), 53.9 (*SE* = 22.2) and 12.7 (*SE* = 6.9) responses per minute. A three-way ANOVA with the factors of stimulus (CS + or CS–) congruence (whether the final discrimination was the same or different to that for training), and discrimination (whether the discrimination was 20+/5– or 5+/20–) indicated that there was a significant effect of stimulus, *F*(1, 12) = 81.00, *p* < .001, but found no significant differences based on discrimination, *F* < 1, or congruence, *F* < 1, and no significant Discrimination × Congruence, *F*(1, 12) = 1.03, *p* > .10, Stimulus × Discrimination, *F*(1, 12) = 2.16, *p* > .10, Stimulus × Congruence, *F* < 1, or thee-way interaction, *F* < 1.

**Figure 9 fig9:**
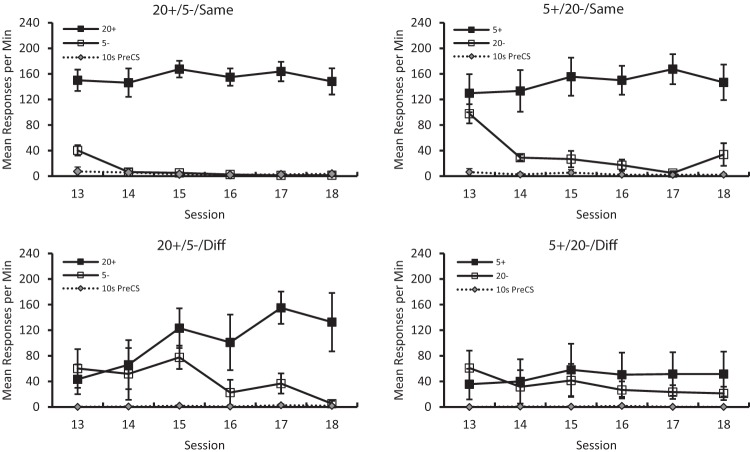
The mean rates of responding to conditioned stimuli (CS) CS+ and CS– for the 20+/5/Same (top left panel), 5+/20–/Same (top right panel), 20+/5–/Different (bottom left panel) and the 5+/20–/Different (bottom right panel) groups during Stage II of Experiment 4. Error bars represent ± *SEM.*

The top panels of Figure 9 show the results during Stage II for the 20+/5–/Same and the 5+/20–/Same groups, who received the same discriminations, in terms of the relationship between the number of squares and the outcomes they signaled, in both stages. It is evident that changing from black squares on a white background, to white squares on a black background had rather little impact on the 20+/5– discrimination and the 5+/20– discrimination. Conversely, when the new discrimination was the opposite of that administered in the initial training, the acquisition of the new task was slower. The bottom left-hand panel indicates that the 20+/5–/Diff group, which was trained with the 5+/20– discrimination and then transferred to the 20+/5– discrimination found it difficult to acquire the new task, as compared with the 20+/5–/Same group. Likewise, the bottom right-hand panel indicates that the 5+/20–/Diff group, which was trained with the 5+/20– discrimination and then transferred to the 20+/5– discrimination, found the new task considerably more difficult than the 5+/20–/Same group.[Fig-anchor fig9]

The results from the experiment were analyzed with a 4-way ANOVA with the factors of Stage-II discrimination (20+/5– or 5+/20–), congruence (whether the Stage–II discrimination was the same or different to the original discrimination), stimulus (CS+ or CS–) and session. The analysis revealed that the four-way interaction was not significant, *F* < 1, but there was a significant Stimulus × Congruence interaction, *F*(1, 12) = 30.39, *p* < .001, η_p_^2^ = .72, 95% CI [.31, .83] which indicates that the discrimination was acquired more readily by the groups receiving the same discrimination in both stages than those receiving different discriminations in both stages. To explore this interaction further, tests of simple main effects were conducted. These tests revealed a significant difference between the overall rates of responding to CS + and CS– for the two groups trained with the same discriminations in both stages, *F*(1, 12) = 123.01, *p* < .001, η_p_^2^ = .91, 95% CI [.72, .95] and for the two groups trained with different discriminations in both stages, *F*(1, 12) = 10.86, *p* = .006, η_p_^2^ = .48, 95% CI [.05, .69]. In addition, there was a significant effect of congruence for CS+, *F*(1, 24) = 20.09, *p* < .001, η_p_^2^ = .46, 95% CI [.15, .64], but not CS–, *F* < 1.

To return to Figure 9, the asymmetry revealed in the previous experiments was again evident in the present study. Thus the discrimination by the 20+/5–/Same group was acquired more readily than by the 5+/20–/Same group, and the discrimination by the 20+/5–/Diff group was acquired more readily than by the 5+/20–/Diff group. In support of these observations, the four-way ANOVA also revealed a significant Stimulus × Session × Stage-II Discrimination interaction, *F*(5, 60) = 5.33, *p* < .001, η_p_^2^ = .31, 95% CI [.08, .43].

The remaining results from the four-way ANOVA were as follows. The effects of session, *F*(5, 60) = 2.17, *p* = .069, congruence, *F*(1, 12) = 4.12, *p* = .065, group, *F*(1, 12) = 1.24, *p* > .10, and did not reach significance, but there was a significant effect of stimulus, *F*(1, 12) = 103.48, *p* < .001, η_p_^2^ = .90, 95% CI [.68, .94]. There were significant interactions of Stimulus × Session, *F*(5, 60) = 27.13, *p* < .001, η_p_^2^ = .69, 95% CI [.53, .76], but the remaining interactions were not significant. Stimulus × Session × Congruence, *F*(5, 60) = 2.12, *p* > .10, Session × Congruence × Stage–II Discrimination, *F* < 1, Session × Stage–II Discrimination, *F* < 1, Session × Congruence, *F*(5, 60) = 1.65, *p* > .10, Stimulus × Congruence × Stage–II Discrimination, *F*(1, 12) = 4.08, *p* = .07, Congruence × Stage–II Discrimination, *F* < 1, and Stimulus × Stage–II Discrimination, *F* < 1.

Figure 9 also reveals that pigeons in all groups made very few responses during the pre-CS period. Comparisons between the mean rates of responding across the six sessions revealed that there were no significant differences between the 20+/5–/Same and 20+/5–/Diff groups, *U*(4, 4) = 6, *p* > .10, or the 5+/20–/Same and 5+/20–/Diff groups, *U*(4, 4) = 5.5, *p* > .10. Additionally, when data was combined across Stage–II discrimination (20+/5– vs. 5+/20–) there was no significant effect of congruence, *U*(4, 4) = 25, *p* > .10, while when the data were combined across congruence there was no effect of Stage–II discrimination, *U*(4, 4) = 27.5, *p* > .10.

### Discussion

The superior Stage–II performance by the groups receiving the same discrimination in both stages, relative to the groups receiving different discriminations, indicates there was a degree of generalization from the stimuli used in Stage I to those used in Stage II. This generalization must have been based on the number of squares within each stimulus but, because of the differences between the stimuli used for the two stages, it follows that the dimension representing number was not confined to squares of a specific color. Instead, the dimension of number permitted generalization between similar quantities, even though the characteristics of the objects belonging to those quantities were physically very different. Such a conclusion indicates that the representation of quantity is not tied to the concrete properties of the training stimuli but, instead, can mediate generalization in a more abstract manner. In the absence of further evidence, it is not possible to specify the nature of the abstract manner in which pigeons represent information about different quantities, but we would not want to argue that they do so by means of counting.

In keeping with conclusions drawn from Experiments 3a and 3b, it is hard to explain the present pattern of results if the original discrimination was based on the overall brightness of the training patterns for two reasons. First, the use of the negatives of the training patterns in the final stage ensured that the level of brightness of the patterns in the two stages was very different, and any generalization between the patterns based on absolute levels of illumination would be slight. Second, if the original discrimination was based on the relative levels of brightness of the two classes of pattern, then transfer to the test patterns should have been superior in the two groups trained with the opposite discriminations in the test than the training stages. This prediction follows because for these groups the brighter patterns, and the dimmer patterns, in both stages signaled the same outcome.

## General Discussion

The experiments confirm that the asymmetry observed in magnitude discriminations involving time, stimulus intensity and the length of an object, can also be found with discriminations in which different numbers of identical objects signal the presence or absence of reward. It would thus appear that the benefit of using a large magnitude to signal reward, and a small magnitude to signal the absence of reward, relative to when the opposite is true, may well be a characteristic of magnitude discriminations in general.

The results from Experiment 2 demonstrate for the first time that by manipulating the stimulation during the ITI it is possible to reverse the asymmetry that is normally found with magnitude discriminations. Thus, rendering the ITI more similar to the larger of the two training stimuli resulted in the 5+/20– discrimination being acquired more readily than 20+/5–. This pattern of results is entirely in keeping with the proposal of Kosaki et al. (2013) that inhibition generalizing from the stimuli present during the ITI to CS+ and CS– is responsible for the asymmetry observed with magnitude discriminations.

A clear prediction that can be drawn from the proposals of Kosaki et al. (2013) is that an asymmetry in a magnitude discrimination will be observed only if there is an opportunity for cues, other than those on which the discrimination is based, to enter into an inhibitory association. This requirement was met in the present experiments. It was also met in the experiments by Kosaki et al. (2013), in which rats had to choose between long and short black panels in order to find a goal. The panels were pasted to the gray walls of a square pool and any approach to the walls by themselves necessarily resulted in failure to find the goal and thus the opportunity for inhibitory conditioning. The requirement was also met in the demonstration of an asymmetry in the discrimination of magnitude using auditory cues by Zielinski and Jakubowska (1977). The method of training was conditioned suppression in which protracted periods of nonreinforced exposure to the background cues separated presentations of the loud and soft auditory cues. The results from experiments by Bouton and Hendrix (2011) and Bouton and García-Gutíerrez (2006), however, do not fit so comfortably with the proposals of Kosaki et al. (2013). Rats received food after a tone when successive presentations of this stimulus were separated by a long, but not a short interval (long+/short-), or they received the opposite of this treatment (short+/long-). Despite the fact that cues indicating the trial outcome were present throughout the experimental session, and there was thus no effective ITI, an asymmetry was still observed: long+/short-discriminations were acquired more readily than the short+/long-discriminations. According to the proposals of Kosaki et al. (2013) this asymmetry should not have been observed as there was no opportunity for background cues, by themselves, to enter into inhibitory associations, and thus no reason to suppose that generalization of inhibition would influence one discrimination more than the other. It remains to be seen whether this challenge to the proposals of Kosaki et al. (2013) means their proposals are wrong, or whether there is more than one mechanism that is responsible for the asymmetry in the discrimination of magnitude.

The experiments have shown that any generalization of inhibition that does take place is unlikely to be based solely on the dimension of brightness. Inspection of the stimuli used for Experiment 1, which are sketched in Figure 1, reveals that they can readily be ordered in ascending order of brightness starting with the 20-square pattern, then the 5-square pattern, and finally the display for the ITI. The results from Experiment 1 alone thus imply that the generalization of inhibition took place along the basic, physical dimension of brightness. Of course, the results from Experiment 3a serve as a forceful counter to this analysis, because changing the stimulation during the ITI from white to black did not reverse the asymmetry, which should have been the case if the magnitude discrimination was based on differences in brightness.

The results from Experiment 3a, as well as those from Experiments 3b and 4, thus led us to conclude that any generalization among the different stimuli used for the experiments involved a less physical, more abstract, dimension than brightness, such as the number of squares displayed within a pattern. The dimension is described as abstract because Experiment 4 revealed substantial transfer of responding between similar numbers of squares, even though they changed in brightness from white to black (see Dumont et al., 2015).

Referring to the number of squares on the screen is not the only possible way in which the discriminations were solved. Rather than refer to the number of squares on the screen, subjects may have relied on the total area occupied by the squares. Since the area occupied by each square was the same, regardless of the pattern to which it belonged, it then follows that the total area occupied by all the squares in a pattern would provide a suitable dimension for generalization among the patterns. Given the considerable transfer in Experiment 4 between patterns comprising black squares on a white background, and those comprising white squares on a black background, it then follows that the representation of a given area of squares would need to be independent of their color.

There is another way in which the discriminations may have been solved. In each experiment, the manner in which the squares were presented ensured that the more squares that were displayed, the smaller was the distance between them. The discriminations may, therefore, have been solved by subjects referring to the distance between the squares, rather than the number of squares. With this possibility in mind, it is noteworthy that the mean distance between adjacent squares was 10 mm, 6 mm, and 4 mm for the patterns containing five, 20, and 288 squares, respectively. Experiment 2 revealed that the birds responded rapidly during a 20-square pattern, and hardly at all during the ITI when 288 squares were presented. Although possible, it seems unlikely that this success was gained solely by the animals distinguishing between the distances of 4 and 6 mm that separated the squares in the different patterns.

The way in which the discriminations were solved is obviously important for research directed at the question of how pigeons are sensitive to differences in quantity but, for present purposes, it does not matter which of the above alternatives is correct. Even if the discriminations were solved by referring to the distance between squares, or the area occupied by squares, the results would still be compatible with the conclusion that there is an asymmetry in the discrimination of magnitude, but now the dimension of magnitude would be the distance between objects, or the area of the objects. Viewing the results in this way does not alter the important empirical conclusion we wish to draw—that there is an asymmetry in discriminations based on the number of objects. Instead, it would suggest that our understanding of how information is encoded about different numbers of the same object is incomplete.

Thus far the explanation for the results in terms of stimulus generalization has been derived from an informal account put forward by Kosaki, Jones and Pearce (2013) which, itself, was based on Spence’s (1936, 1937) account of discrimination learning. Is it possible to explain the results more formally in terms of more recent theories of learning? A challenge posed by this question is that to our knowledge a formal account of stimulus generalization along a dimension of magnitude does not exist. One way of addressing this problem is to follow the proposals of Bouton and Hendrix (2011) concerning the way in which animals solve discriminations between stimuli of different durations. In essence, they suggested that a short duration stimulus is composed of one element, say A, and a long duration stimulus is composed of a succession of elements, say A followed by B. As far as the present experiments are concerned, it might then be suggested that the dimension of number can be represented by an increasing number of distinctive elements. The dimension would be anchored by one element, A, to represent zero, two elements, AB, to represent a trial with 5 squares, three elements, ABC, to represent a trial with 20 squares, and four elements ABCD, to represent 288 squares present during the ITI. Using this characterization it is then possible to apply current theories of learning to the present experiments.

According to the theory of Pearce (1994), for example, the presence of the common element A, will permit generalization among the three patterns (the two training stimuli and the ITI) in any experiment, with generalization being greater for patterns representing magnitudes that are close together. Computer simulations based on equations proposed by Pearce (1994) indicated that the theory is able to predict the asymmetry in the magnitude discriminations that was observed in every experiment. A further set of simulations was conducted by using the equation proposed by Rescorla and Wagner (1972) to predict the changes in associative strength of individual elements in the training patterns described above. Once again, the asymmetry observed in all of the experiments was predicted successfully but, in the case of Experiment 2, not entirely satisfactorily. During the early stages of training the 20+/5– discrimination was incorrectly predicted to be acquired more readily than 5+/20–; it was only in the later stages that this relationship was predicted to reverse.

For all of the above simulations, in keeping with arguments put forward by Rescorla and Wagner (1972) and Jones and Pearce (2015), the value for the learning rate parameter, β, which plays the same role in both theories, was greater for reinforced than nonreinforced trials. If the same value of β is used for the reinforced and nonreinforced trials then the Rescorla-Wagner equation predicts quite well the results from Experiment 2, as well as the other experiments. Varying the values of β for the reinforced and nonreinforced trials also influences predictions derived from the theory of Pearce (1994). The asymmetry that was observed in each experiment continues to be correctly predicted by the theory, but the magnitude of the asymmetry becomes smaller as the value of β for reinforced trials decreases and that for the nonreinforced trials increases.

An unexpected finding from the experiments was the failure of the 20+/5– group in Experiment 2 to solve its discrimination. In this experiment, the TV screen during the ITI displayed 288 squares. All of the simulations that have just been described for both theories predict this discrimination should have been acquired readily, irrespective of the values assigned to β. It remains to be determined whether the failure to confirm this prediction is indicative of a serious shortcoming with the explanations offered by these theories for our results.

An intriguing feature of the above discussion is that in order to explain our results it is necessary to assume that when no squares are on the screen, the consequent stimulation is represented as zero on a dimension of number. In order to permit generalization from trials with zero squares to trials with 5 or 20 squares, it was further necessary to assume that zero is represented by an element that is also activated when one or more squares are portrayed on the screen. Apart from being able to explain the present results, it is hard to think of any additional justification for these assumptions. In view of the slender support for them, therefore, it might be prudent not to abandon the search for an alternative to the foregoing explanations for our findings.
